# HALL: a comprehensive database for human aging and longevity studies

**DOI:** 10.1093/nar/gkad880

**Published:** 2023-10-23

**Authors:** Hao Li, Song Wu, Jiaming Li, Zhuang Xiong, Kuan Yang, Weidong Ye, Jie Ren, Qiaoran Wang, Muzhao Xiong, Zikai Zheng, Shuo Zhang, Zichu Han, Peng Yang, Beier Jiang, Jiale Ping, Yuesheng Zuo, Xiaoyong Lu, Qiaocheng Zhai, Haoteng Yan, Si Wang, Shuai Ma, Bing Zhang, Jinlin Ye, Jing Qu, Yun-Gui Yang, Feng Zhang, Guang-Hui Liu, Yiming Bao, Weiqi Zhang

**Affiliations:** CAS Key Laboratory of Genomic and Precision Medicine, Beijing Institute of Genomics, Chinese Academy of Sciences and China National Center for Bioinformation, Beijing 100101, China; University of Chinese Academy of Sciences, Beijing 100049, China; CAS Key Laboratory of Genome Sciences and Information, Beijing Institute of Genomics, Chinese Academy of Sciences and China National Center for Bioinformation, Beijing 100101, China; National Genomics Data Center, Beijing Institute of Genomics, Chinese Academy of Sciences and China National Center for Bioinformation, Beijing 100101, China; University of Chinese Academy of Sciences, Beijing 100049, China; CAS Key Laboratory of Genomic and Precision Medicine, Beijing Institute of Genomics, Chinese Academy of Sciences and China National Center for Bioinformation, Beijing 100101, China; University of Chinese Academy of Sciences, Beijing 100049, China; Interdisciplinary Institute for Medical Engineering, Fuzhou University, Fuzhou 350002, China; CAS Key Laboratory of Genomic and Precision Medicine, Beijing Institute of Genomics, Chinese Academy of Sciences and China National Center for Bioinformation, Beijing 100101, China; University of Chinese Academy of Sciences, Beijing 100049, China; Sino-Danish College, University of Chinese Academy of Sciences, Beijing 101408, China; Department of Vascular Surgery, Quzhou Affiliated Hospital of Wenzhou Medical University, Beijing 100101, China; The Joint Innovation Center for Engineering in Medicine, Quzhou Affiliated Hospital of Wenzhou Medical University, 324000, China; CAS Key Laboratory of Genomic and Precision Medicine, Beijing Institute of Genomics, Chinese Academy of Sciences and China National Center for Bioinformation, Beijing 100101, China; University of Chinese Academy of Sciences, Beijing 100049, China; Sino-Danish College, University of Chinese Academy of Sciences, Beijing 101408, China; Institute for Stem Cell and Regeneration, Chinese Academy of Sciences, Beijing 100101, China; CAS Key Laboratory of Genomic and Precision Medicine, Beijing Institute of Genomics, Chinese Academy of Sciences and China National Center for Bioinformation, Beijing 100101, China; University of Chinese Academy of Sciences, Beijing 100049, China; CAS Key Laboratory of Genomic and Precision Medicine, Beijing Institute of Genomics, Chinese Academy of Sciences and China National Center for Bioinformation, Beijing 100101, China; University of Chinese Academy of Sciences, Beijing 100049, China; CAS Key Laboratory of Genomic and Precision Medicine, Beijing Institute of Genomics, Chinese Academy of Sciences and China National Center for Bioinformation, Beijing 100101, China; University of Chinese Academy of Sciences, Beijing 100049, China; CAS Key Laboratory of Genomic and Precision Medicine, Beijing Institute of Genomics, Chinese Academy of Sciences and China National Center for Bioinformation, Beijing 100101, China; University of Chinese Academy of Sciences, Beijing 100049, China; CAS Key Laboratory of Genomic and Precision Medicine, Beijing Institute of Genomics, Chinese Academy of Sciences and China National Center for Bioinformation, Beijing 100101, China; University of Chinese Academy of Sciences, Beijing 100049, China; CAS Key Laboratory of Genomic and Precision Medicine, Beijing Institute of Genomics, Chinese Academy of Sciences and China National Center for Bioinformation, Beijing 100101, China; University of Chinese Academy of Sciences, Beijing 100049, China; Department of Vascular Surgery, Quzhou Affiliated Hospital of Wenzhou Medical University, Beijing 100101, China; CAS Key Laboratory of Genomic and Precision Medicine, Beijing Institute of Genomics, Chinese Academy of Sciences and China National Center for Bioinformation, Beijing 100101, China; University of Chinese Academy of Sciences, Beijing 100049, China; CAS Key Laboratory of Genomic and Precision Medicine, Beijing Institute of Genomics, Chinese Academy of Sciences and China National Center for Bioinformation, Beijing 100101, China; University of Chinese Academy of Sciences, Beijing 100049, China; CAS Key Laboratory of Genomic and Precision Medicine, Beijing Institute of Genomics, Chinese Academy of Sciences and China National Center for Bioinformation, Beijing 100101, China; University of Chinese Academy of Sciences, Beijing 100049, China; Department of Vascular Surgery, Quzhou Affiliated Hospital of Wenzhou Medical University, Beijing 100101, China; Advanced Innovation Center for Human Brain Protection, and National Clinical Research Center for Geriatric Disorders, Xuanwu Hospital Capital Medical University, Beijing 100053, China; Aging Translational Medicine Center, Xuanwu Hospital, Capital Medical University, Beijing 100053, China; Advanced Innovation Center for Human Brain Protection, and National Clinical Research Center for Geriatric Disorders, Xuanwu Hospital Capital Medical University, Beijing 100053, China; Aging Translational Medicine Center, Xuanwu Hospital, Capital Medical University, Beijing 100053, China; Aging Biomarker Consortium, Beijing 100101, China; State Key Laboratory of Membrane Biology, Institute of Zoology, Chinese Academy of Sciences, Beijing 100101, China; University of Chinese Academy of Sciences, Beijing 100049, China; Institute for Stem Cell and Regeneration, Chinese Academy of Sciences, Beijing 100101, China; Aging Biomarker Consortium, Beijing 100101, China; CAS Key Laboratory of Genomic and Precision Medicine, Beijing Institute of Genomics, Chinese Academy of Sciences and China National Center for Bioinformation, Beijing 100101, China; Department of Vascular Surgery, Quzhou Affiliated Hospital of Wenzhou Medical University, Beijing 100101, China; State Key Laboratory of Stem Cell and Reproductive Biology, Institute of Zoology, Chinese Academy of Sciences, Beijing 100101, China; University of Chinese Academy of Sciences, Beijing 100049, China; Beijing Institute for Stem Cell and Regenerative Medicine, Beijing 100101, China; Institute for Stem Cell and Regeneration, Chinese Academy of Sciences, Beijing 100101, China; Aging Biomarker Consortium, Beijing 100101, China; CAS Key Laboratory of Genomic and Precision Medicine, Beijing Institute of Genomics, Chinese Academy of Sciences and China National Center for Bioinformation, Beijing 100101, China; University of Chinese Academy of Sciences, Beijing 100049, China; Sino-Danish College, University of Chinese Academy of Sciences, Beijing 101408, China; The Joint Innovation Center for Engineering in Medicine, Quzhou Affiliated Hospital of Wenzhou Medical University, 324000, China; State Key Laboratory of Membrane Biology, Institute of Zoology, Chinese Academy of Sciences, Beijing 100101, China; University of Chinese Academy of Sciences, Beijing 100049, China; Beijing Institute for Stem Cell and Regenerative Medicine, Beijing 100101, China; Institute for Stem Cell and Regeneration, Chinese Academy of Sciences, Beijing 100101, China; Advanced Innovation Center for Human Brain Protection, and National Clinical Research Center for Geriatric Disorders, Xuanwu Hospital Capital Medical University, Beijing 100053, China; Aging Translational Medicine Center, Xuanwu Hospital, Capital Medical University, Beijing 100053, China; Aging Biomarker Consortium, Beijing 100101, China; CAS Key Laboratory of Genome Sciences and Information, Beijing Institute of Genomics, Chinese Academy of Sciences and China National Center for Bioinformation, Beijing 100101, China; National Genomics Data Center, Beijing Institute of Genomics, Chinese Academy of Sciences and China National Center for Bioinformation, Beijing 100101, China; University of Chinese Academy of Sciences, Beijing 100049, China; CAS Key Laboratory of Genomic and Precision Medicine, Beijing Institute of Genomics, Chinese Academy of Sciences and China National Center for Bioinformation, Beijing 100101, China; University of Chinese Academy of Sciences, Beijing 100049, China; Sino-Danish College, University of Chinese Academy of Sciences, Beijing 101408, China; Institute for Stem Cell and Regeneration, Chinese Academy of Sciences, Beijing 100101, China; Aging Biomarker Consortium, Beijing 100101, China

## Abstract

Diverse individuals age at different rates and display variable susceptibilities to tissue aging, functional decline and aging-related diseases. Centenarians, exemplifying extreme longevity, serve as models for healthy aging. The field of human aging and longevity research is rapidly advancing, garnering significant attention and accumulating substantial data in recent years. Omics technologies, encompassing phenomics, genomics, transcriptomics, proteomics, metabolomics and microbiomics, have provided multidimensional insights and revolutionized cohort-based investigations into human aging and longevity. Accumulated data, covering diverse cells, tissues and cohorts across the lifespan necessitates the establishment of an open and integrated database. Addressing this, we established the Human Aging and Longevity Landscape (HALL), a comprehensive multi-omics repository encompassing a diverse spectrum of human cohorts, spanning from young adults to centenarians. The core objective of HALL is to foster healthy aging by offering an extensive repository of information on biomarkers that gauge the trajectory of human aging. Moreover, the database facilitates the development of diagnostic tools for aging-related conditions and empowers targeted interventions to enhance longevity. HALL is publicly available at https://ngdc.cncb.ac.cn/hall/index.

## Graphical Abstract

**Graphical Abstract:**
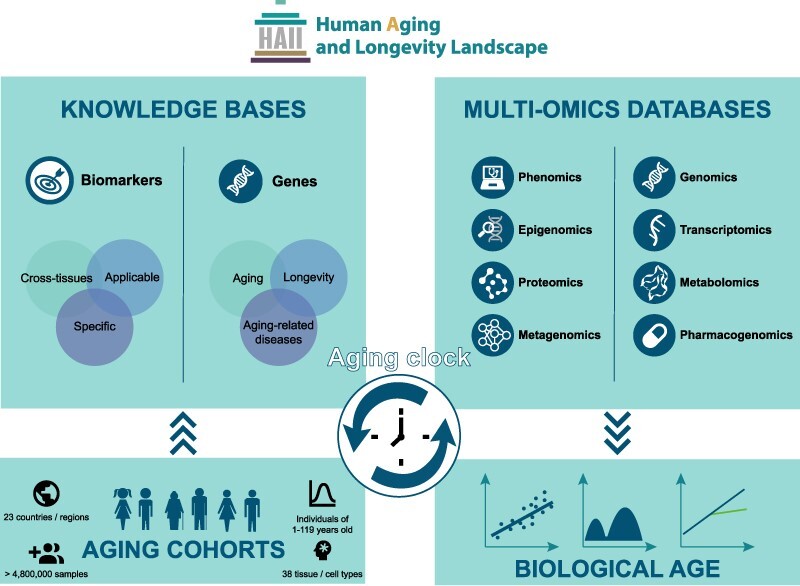

## Introduction

As we transition through different stages of life, from childhood to adulthood and ultimately old age, we undergo substantial transformations and reach major milestones (1,2). Centenarians, in particular, have emerged as potential models for healthy aging, as they exemplify extreme longevity (3–5). However, it is important to recognize that individuals age at different rates and exhibit varying susceptibility to tissue aging, functional decline and aging-related diseases (6–8). Therefore, there is a pressing need for a comprehensive framework that can serve as a benchmark for monitoring human age-related changes (8–11). The study of human aging and longevity is a rapidly evolving field that has garnered significant attention and accumulated tremendous amounts of data in recent years (12–16). Omics technologies, including phenomics, genomics, transcriptomics, proteomics, metabolomics and microbiomics, have transformed the study of human aging. These technologies provide multi-dimensional insights and have also revolutionized cohort-based research on understanding human aging and longevity (17–19). The accumulation of these valuable data from diverse cells, tissues, and cohorts across the lifespan necessitates the establishment of an open and integrated database.

Currently, there is a lack of established multi-modal databases that focus on aggregating omics data spanning human tissues, cohorts and age ranges, with a specific emphasis on data related to human aging and longevity. Existing databases, such as the Human Genome Resources at NCBI and Genome Sequence Archive for Human, are not tailored for the need to investigate human aging and longevity biomarkers and sensitivity to aging-related diseases. Other databases, such as Aging Atlas (20) and Human Aging Genomic Resources (21), offer limited scattered information within their mixed datasets, making it difficult to integrate and analyze human omics data across different studies. Furthermore, previous databases typically only contain data recorded at two time points (i.e. young and old ages), which is insufficient considering that age is a continuous and dynamic process. This limitation restricts their utility in archiving complex alterations during the aging trajectory. (22,23) The HALL database fills this important gap by providing a specialized, well-organized and comprehensive compilation of multi-dimensional datasets obtained from diverse human cohorts, focused on human aging and longevity. By providing a framework for monitoring age-related changes, HALL can facilitate the development of novel biomarkers, diagnostic tools and interventions for aging and aging-related diseases.

To the best of our knowledge, none of the existing databases offer a comprehensive compilation of aging- and longevity-related cohorts and associated studies. The availability of well-established cohorts such as the UK Biobank, Baltimore Longitudinal Study of Aging (BLSA), China Health and Retirement Longitudinal Study (CHARLS), Chinese Longitudinal Healthy Longevity Surveys (CLHLS), Framingham Heart Study (FHS) and National Health and Nutrition Examination Survey (NHANES), have supported the publication of thousands of scientific papers and made significant contributions to our understanding of aging, longevity and related diseases (24–32). Moreover, with the global aging population, an increasing number of cohorts supporting omics-based research have been established worldwide (33–36). However, there is a noticeable gap in the aggregation of these published studies, preventing comprehensive investigations and comparisons of the determinants of aging and longevity across different cohorts and countries. In addition, the recent rapid development of artificial intelligence has made it possible to use this collection of human datasets to develop the ‘digital twins’ capable of modeling one's health status, which might be a promising approach to achieving personalized medicine and promoting lifelong health (37–39).

We here present the Human Aging and Longevity Landscape (HALL), a comprehensive and multi-modal web resource for the exploration of human physiology and pathology in all stages of the lifespan. Guided by the Aging Biomarker Consortium (ABC), HALL is a unique and valuable resource that fills a critical gap in the field of aging and longevity research. All the data are manually collected from the literature and retrieved from existing databases, providing a comprehensive and reliable source of multi-dimensional datasets.

## Database contents

**Figure 1.**
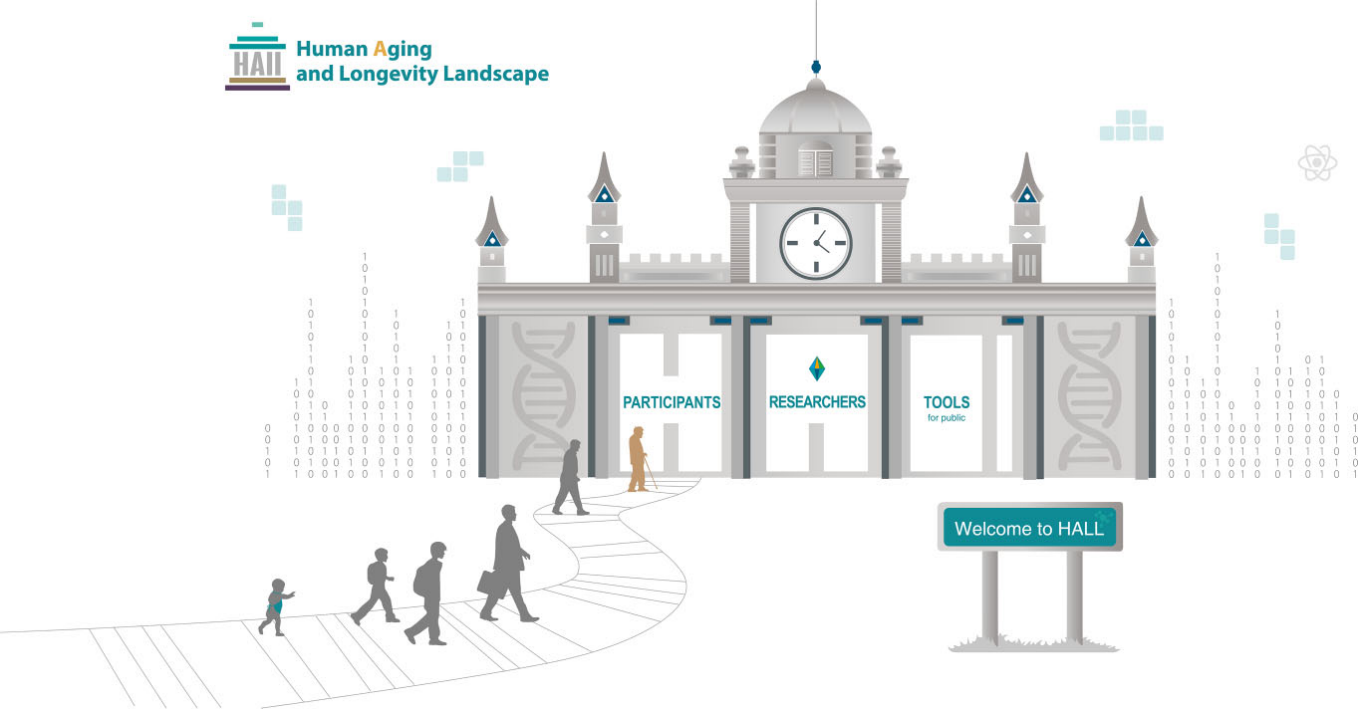
The HALL database comprises three interlinked and symbiotic components: HALL for Researchers, Participants and the Public.

**Figure 2.**
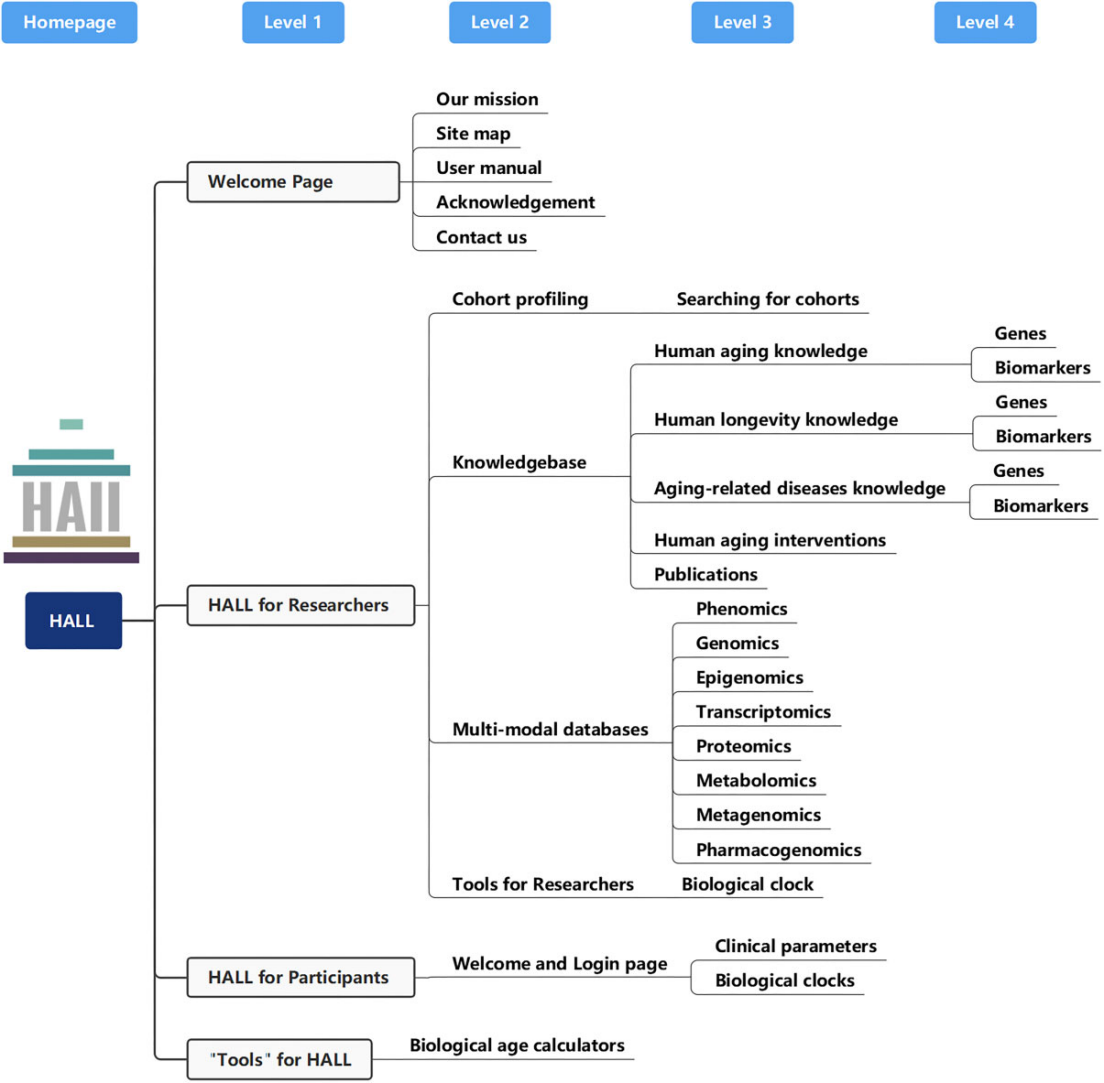
The site map of HALL, illustrating the hierarchical structure (from the homepage to levels 1, 2, 3, 4) of the platform and guiding users to navigate through the different levels of HALL.

The HALL database consists of three interconnected and mutually supportive components: HALL for Researchers, Participants and the Public (Figures 1 and 2). The primary focus is HALL for researchers, which offers open access to comprehensive and multi-dimensional datasets sourced from diverse human cohorts. Additionally, it serves as an open-access knowledgebase, providing meticulously curated information on aging- and longevity-associated genes, biomarkers and biological age clocks. HALL for participants enables volunteers to access and view their own information with assigned accounts. The 'Tools' section is built for the public, offering established biological clocks that utilize clinically available parameters. Thus, the HALL database offers unique knowledge and insights that can support scientific research, enhance clinical practice and fuel industry initiatives in the field of human physiology and pathology, particularly in the areas of aging, aging-related diseases and longevity.

## HALL for researcher

**Figure 3.**
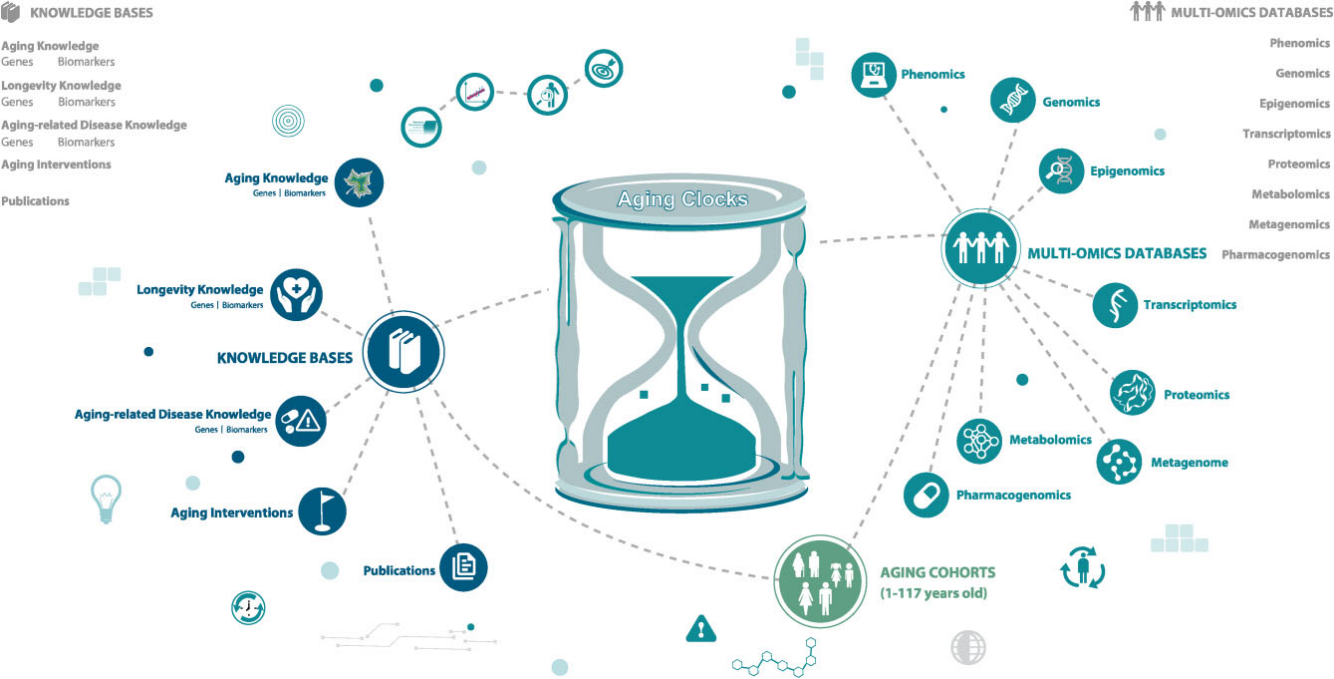
Homepage of HALL for Researchers. This section consists of three major components: Cohort Information, Knowledge Curation and Multi-omics Analysis. As demonstrated by the diagram at the center of the page, HALL for Researchers collects curated knowledge and datasets from various cohorts and previous literature, providing a rich resource for the identification of aging biomarkers and potential interventional targets. This integrated resource can further facilitate the establishment of aging clocks from different aspects of health that are capable of measuring biological age, which might differ between centenarians and non-centenarians.

The researchers’ platform of the Human Aging and Longevity Landscape (**HALL for Researchers**) is dedicated to providing a wealth of data and information that enables researchers to explore and analyze the phenotypic and molecular changes and biomarkers associated with human aging and longevity (Figure 3). By accessing this platform, researchers from various fields can delve into the intricate mechanisms underlying human aging, gain valuable insights into the factors that influence longevity and investigate potential interventions and strategies for promoting healthy aging. HALL for Researchers encompasses a series of modules, fortified to serve as an encyclopedic and omics resource for research on aging and longevity (Figure 3).

### Cohort profile: overview of major population studies in the HALL database

In the HALL project, we present a concise overview of cohort studies encompassing a broad age range and diverse populations. Our database comprises both large-scale and small-scale cohorts from various regions and countries, representing both genders and diverse ethnicities. Notable cohort studies included in the database are the UK Biobank (UK), the Framingham Heart Study (USA), Chinese Longitudinal Healthy Longevity Surveys (China), National Health and Nutrition Examination Survey (USA), European Network for Genetic and Genomic Epidemiology (Europe), and InCHIANTI study (Italy). In total, we have incorporated 170 cohorts from 23 countries/regions, involving more than 4 800 000 individuals and including 38 different tissue/cell types. These datasets encompass individuals ranging from 1 to 119 years of age, with 59 cohorts containing centenarians. Conducting comparative analyses across these cohorts can enhance our understanding of causal relationships and help identify mediating factors unique to specific cohorts. We are committed to continually expanding the database with additional cohorts in the future, fostering a more comprehensive understanding of the aging process across diverse populations.

### Multi-modal datasets: omics database for aging and longevity studies

The core component of the HALL database, dedicated to researchers and centered on human data, currently includes eight distinct omics data types: phenomics, genomics, epigenomics, bulk and single-cell transcriptomics, proteomics, metabolomics, metagenomics and pharmacogenomics.

### The phenomics module: curating a list of phenotypic features closely associated with human aging and longevity

**Figure 4.**
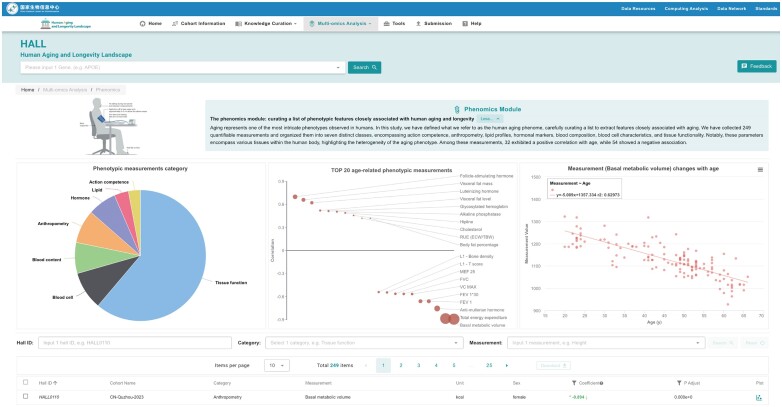
Phenomics Module Page Layout: Divided into three sections - introduction, visualization functions and measurement retrieval.

Aging represents one of the most intricate phenotypes observed in humans. In this study, we have defined what we refer to as the human aging phenome, carefully curating a list to extract features closely associated with aging (Figure 4). We have collected 249 quantifiable measurements and organized them into seven distinct classes, encompassing action competence, anthropometry, lipid profiles, hormonal markers, blood composition and blood cell characteristics. These parameters could also potentially be linked to the functions of diverse tissues within the human body, emphasizing the heterogeneous nature of the aging phenotype. Among these measurements, 32 exhibited a positive correlation with age, while 54 showed a negative association.

### The genomics module: a rich resource to view single nucleotide polymorphisms (SNPs) that are associated with aging or longevity traits

Aging is simultaneously affected by a combination of genetic variations and environmental exposures. Identification of genomic variants related to aging has the potential to reveal fundamental biological processes underlying the dynamics of aging. Large-scale genome-wide association studies (GWAS) have identified many loci related to human aging and longevity. Collecting data from the Literature and GWAS Catalog (40) focusing on aging and longevity, this module offers a comprehensive resource to access and download single nucleotide polymorphisms (SNPs) that are associated with human aging traits, including longevity, healthspan and lifespan. This collection consists of 1913 SNPs sourced from 2 166 953 individuals and 50 cohorts. The age range of individuals involved in these studies spans below 119 years old.

### The epigenomics module: a powerful tool for querying DNA methylation alterations throughout the human lifespan

Increasing evidence suggests a connection between human aging and epigenetic mechanisms (41–43). One prominent characteristic of aging is the presence of global DNA hypomethylation, coupled with hypermethylation at specific CpG islands (41,42). Age estimators, including Horvath's clock, Hannum's clock and DNAm PhenoAge, rely on analyzing DNA methylation changes in the genome. Utilizing the extensive repository of age-related data within EWAS Open Platform and literature (44), this module enables users to inquire about DNA methylation variations throughout the human lifespan. In addition, this module provides a genome browser for 485512 probes, along with the distribution of age and methylation changes.

### The transcriptomics module: integrative RNA-seq datasets of humans in all stages of the lifespan

**Figure 5.**
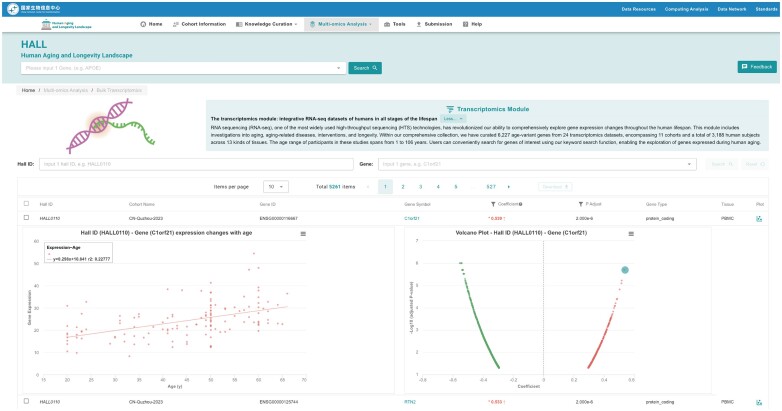
Page of the Transcriptomics module. This page is divided into three regions: the introduction of the module at the top, the visualization region in the middle, and the table for gene retrieval at the bottom. In the table region, the users can search the genes by the gene name and view their association with age. In addition, users can further view the expression levels of a certain gene in individuals across different ages by clicking the gene name.

RNA sequencing (RNA-seq), one of the most widely used high-throughput sequencing (HTS) technologies, has enhanced our ability to comprehensively explore gene expression changes throughout the human lifespan (Figure 5). Within our comprehensive collection, we have curated 5261 age-variant genes from 24 transcriptomics datasets, encompassing 25 cohorts and a total of 3188 human subjects across 13 kinds of tissues. The age range of participants in these studies spans from 1 to 106 years. Users can conveniently search for genes of interest using our keyword search function, enabling the exploration of genes expressed during human aging.

### The single-cell transcriptomics module: a rich resource for the single-cell molecular framework of human tissue and organ aging, and human aging-related diseases

Advancements in single-cell technologies have empowered researchers to generate extensive datasets, enabling profound decoding of human aging with unparalleled scope and resolution (17,45). Focusing on human aging and longevity, this module serves as a systematic documentation of cell type-specific changes in gene expression across multiple human tissues. Although the application of single-cell RNA sequencing has thus far been limited to a few human tissues from cohorts of healthy aging individuals, such as skin and blood cells, we aim to keep a keen focus on this emerging field and provide an overview of these relevant studies.

### The proteomics module: presenting proteome-wide atlases of age-associated alterations in humans

**Figure 6.**
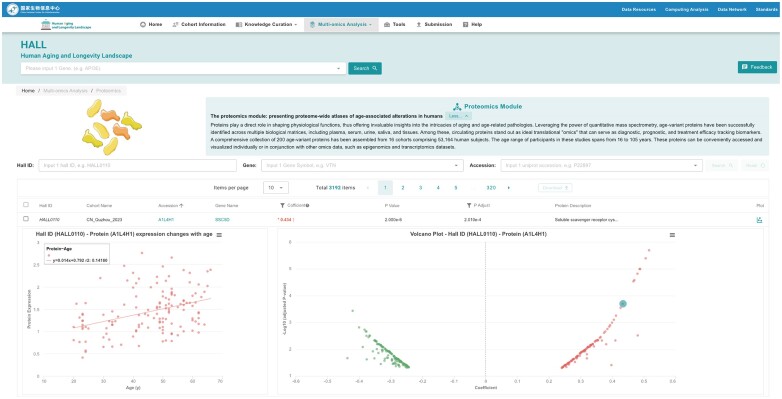
Page of the Proteomics module. This page is divided into three regions: the introduction of the module at the top, the visualization region in the middle, and the table for protein retrieval at the bottom. In the table region, the users can search the proteins by the gene name or UniProt accession and view their association with age. In addition, users can further view the abundance of a certain protein in individuals across different ages by clicking the accession.

Proteins play a direct role in shaping physiological functions, thus offering invaluable insights into the intricacies of aging and age-related pathologies. Leveraging the power of quantitative mass spectrometry, age-variant proteins have been successfully identified across multiple biological matrices, including plasma, serum, urine, saliva and tissues. Among these, circulating proteins stand out as ideal translational ‘omics’ that can serve as diagnostic, prognostic and treatment efficacy tracking biomarkers. A comprehensive collection of 3192 age-variant proteins has been assembled from 16 cohorts comprising 53164 human subjects. The age range of participants in these studies spans from 16 to 105 years. These proteins can be conveniently accessed and visualized individually or in conjunction with other omics data, such as epigenomics and transcriptomics datasets (Figure 6).

### The metabolomics module: presenting metabolome-wide atlases of age-associated alterations in humans

Metabolites are small molecules that are involved in various metabolic processes in the body. By studying the metabolome, which refers to the complete set of metabolites in a biological sample, researchers can gain insights into the biochemical pathways and processes that are affected by aging. This information can help in understanding the physiological changes that occur with age and potentially identify biomarkers or therapeutic targets for age-related conditions. Here, we collected 120 and 110 metabolites that were positively and negatively correlated with age, respectively, based on data from various cohorts.

### The metagenomics module: revealing age- and longevity-related alterations in human microbiota

The human gut microbiome, a complex ecosystem, plays a significant role in the aging process. Metagenomic analyses provide valuable insights into the microbiome's functions that influence human aging and longevity. This module highlights substantial changes in the gut microbiome as age advances, offering a deeper understanding of the interplay between microorganisms and aging, ultimately facilitating targeted interventions for age-related conditions.

### Knowledge curation: resource for aging-related genes, biomarkers and intervention

This part operates as an open-access knowledge base that provides carefully curated information on aging- and longevity-associated genes, biomarkers and knowledge pertaining to aging interventions.

### Aging-related genes and biomarkers: A compilation of human lifespan-related genes, as well as aging biomarkers that serve as indicators of organ and systemic aging

This section encompasses two features: ‘Genes’, which compiles genes associated with human aging that are sourced from literature and annotated. Furthermore, the section incorporates an assemblage of ‘Biomarkers’, according to the suggested criteria of the American Federation for Aging Research. These criteria encompass the biomarker's prognostic potential for the aging pace, its capacity to monitor fundamental processes intrinsic to aging rather than disease effects, its suitability for repeated non-invasive testing, and its applicability in both human subjects and laboratory animals.

### Longevity-related genes and biomarkers: a compilation of human longevity-related genes, as well as longevity biomarkers

This section encompasses two components: ‘Genes’, compiling human lifespan-related genes with pathway annotations, underlining the genetic influence on longevity. Notably, extreme longevity in humans exhibits a strong genetic underpinning. Large-scale GWAS investigations have successfully identified over 51 genetic loci associated with longevity, underscoring the polygenic nature of human lifespan regulation. ‘Biomarkers,’ will present biomarkers of healthy aging and longevity, and a biomarker of longevity and healthy aging essentially (i) discriminates individuals based on their familial propensity for longevity, associates with (ii) known health parameters, and (iii) morbidity and/or mortality in prospective studies and (iv) shows a change with chronological age.

### Aging-related diseases-associated genes and biomarkers: human aging-related disease knowledgebase, with associated genes and diagnostic biomarkers

Aging is the leading risk factor for numerous diseases and conditions that categorizes into distinct groups, such as neurodegenerative diseases, cardiovascular diseases, metabolic diseases and more. This is an integrated database of aging-related conditions and their annotations, and the site allows aging-related disease genes to be viewed and analyzed in the friendly interface. The comprehensive compilation of HALL comprises 52 disease entries consolidated from 9 diverse categories. This section provides an inventory of associated genes for specific aging-related diseases. The gene list comprises resources on genetic variations, offering causative variations specific to the disease, along with genes manually curated for their associations with the disease. Users can search for relevant genes and publications. HALL also includes a collection of biomarkers that are either currently utilized clinically or show strong potential for clinical use in various aging-related diseases. These biomarkers play a crucial role in the diagnosis, prognosis, and management of these age-associated conditions.

### Aging intervention strategies: a toolkit for alleviating aging and aging-related diseases

Within this section, we present a concise summary of a wide array of strategies devised to combat aging and its associated diseases. These strategies encompass small molecule compounds, gene therapies, senolytics, stem cell therapies and proactive health measures such as dietary interventions, exercise and circadian rhythm regulation. While a majority of these interventions are still grounded in animal model research, several advanced intervention methods have entered the clinic and laid the foundation for practical applications. Those strategies with evidence in humans are highlighted in our database and the relevant studies can be redirected by clicking the name of interventions.

### Publications: an integrated knowledge base of publications related to human aging and longevity

Utilizing ‘Human,’ ‘Aging’ and ‘Longevity’ as keywords, we compiled a comprehensive collection of 2724 publications published within the past two decades. This curated list will continue to be regularly updated to incorporate influential papers within the field.

### Data upload and feedback

For users who wish to submit data to this database, we added a data upload feature. Users can submit data by providing their email and properly formatted omics data. User feedback is immensely helpful for optimizing the website. We developed a feedback function, and upon submitting feedback, we promptly review and make necessary improvements.

## HALL for participants

The Participants Platform of the Human Aging and Longevity Landscape enables volunteers participating in registered clinical trials to access a system that provides information about their age-related characteristics. This includes assessments of their phenotypic age and biological age, which are calculated based on diverse omics data. These metrics serve as valuable references for volunteers, enabling them to better understand their overall bodily functions and health status. Additionally, this section incorporates a feedback mechanism that facilitates communication between researchers and volunteers. The data collected through this platform will be utilized, with the volunteers' consent, to expand the Research HALL and further advance the pursuit of knowledge in this field.

## HALL for public

**Figure 7.**
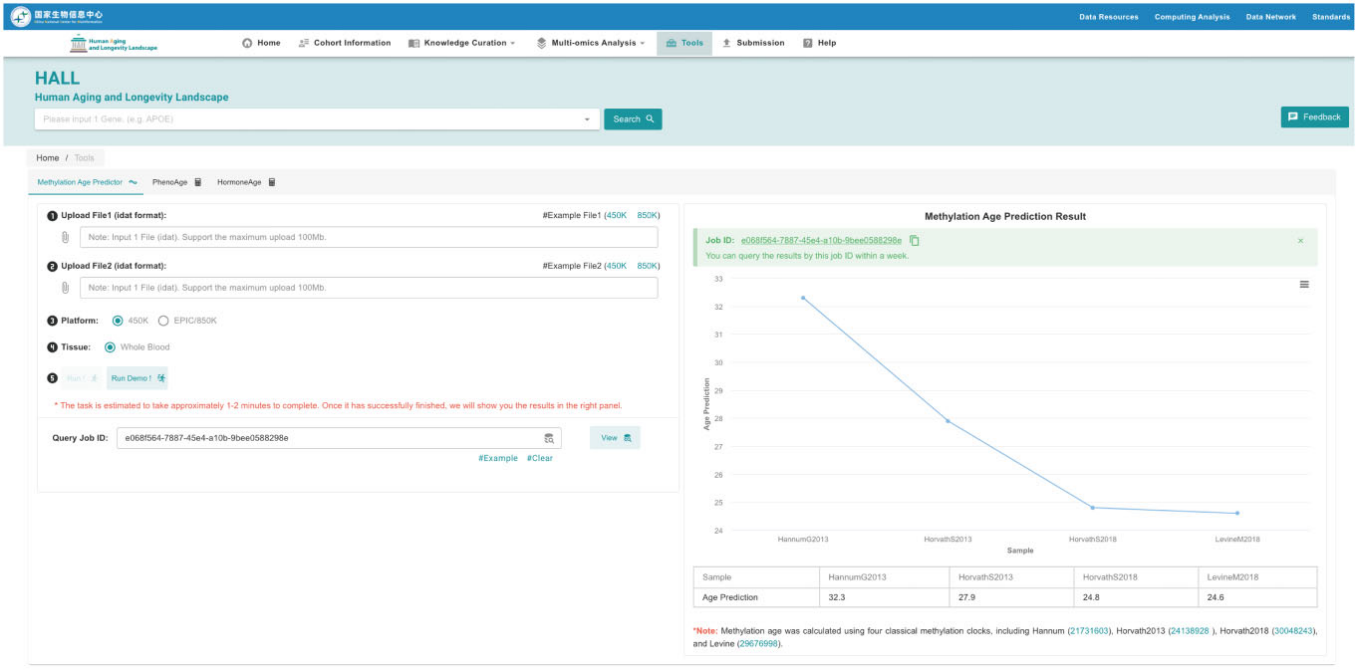
Page of Methylation Age Predictor. The users can upload their DNA methylation probe data, and after computation, receive predictions of different DNA methylation clocks to estimate their ages.

The ‘**Tools**’ section of HALL aims to provide accessible biological age calculators to the public. Biological age refers to the rate at which the body undergoes degeneration and the entropy of its tissues. Our vision is to leverage the abundant data from HALL to expand our range of tools for assessing biological age, encompassing various bodily systems such as the nervous system and blood circulatory system, among others. Currently, we have implemented a phenotypic age and hormone age clock that only requires the users to fill out a single format and can provide insights into how their body functions in relation to their chronological age. We have also developed a DNA methylation clock web tool, which enables users to calculate DNA methylation age online by uploading DNA methylation probe data. Currently, whole blood is the only available option. It is important to note that due to the intricacies of individual health states, this tool serves solely as an advisory tool to assess a person's biological age (Figure 7).

## Concluding remarks

HALL is the pioneering database dedicated to gathering datasets from diverse cohorts focused on aging and longevity. HALL archives both large- and small-scale cohorts encompassing a wide range of populations, including both genders and diverse ethnicities. Notable cohorts include the UK Biobank, CLHLS from China, BLSA from USA, and the CN-Quzhou-2023 cohort established by our group. Moreover, the HALL database provides users with the ability to perform cross-cohort analyses, allowing for the identification of common aging-related features and differences across different populations and regions. HALL covers eight omics data types, accumulating 11256 aging-related features and biomarkers across 38 tissue/cell types. This platform empowers the exploration of aging- and longevity-related changes and the identification of the associations between multi-layered traits and aging dynamics. Last, HALL’s integration of Participants, Researchers and Tools creates an interconnected system supporting aging and longevity research for various stakeholders. This linkage enables data integration across sources, aiding biomarker research and intervention target identification. We are committed to continually updating the HALL database with high-quality aging- and longevity-related omics data and improved functionalities. As a result, our database will continue to provide a valuable resource for the clinical community and life scientists more broadly.

## Data Availability

HALL is publicly available at https://ngdc.cncb.ac.cn/hall/index.
